# De Novo Designed β‐Hairpin Peptides Mimicking the Copper‐Binding Histidine Brace Motif of Lytic Polysaccharide Monooxygenases

**DOI:** 10.1002/anie.202513990

**Published:** 2025-08-15

**Authors:** Enrico Falcone, Rosemary Tomey, Emma Turley, David Cannella, David Robinson, Luisa Ciano

**Affiliations:** ^1^ School of Chemistry University of Nottingham, University Park Nottingham NG7 2RD UK; ^2^ PhotoBiocatalysis Unit, Biomass Transformation Lab – BTL, and Crop Production Biostimulation Lab – CPBL Université Libre de Brussels, ULB Brussels Belgium; ^3^ Department of Chemistry and Forensics School of Science and Technology Nottingham Trent University Nottingham NG11 8NS UK; ^4^ Present address: LCC‐CNRS Université de Toulouse, CNRS Toulouse France

**Keywords:** Copper enzymes, LPMO, Metal‐binding peptides, Polysaccharide degradation

## Abstract

Lytic polysaccharide monooxygenases (LPMOs) are Cu‐containing enzymes that play a crucial role in lignocellulosic biomass degradation for use in biofuel production. These enzymes carry out the selective oxidation of C─H bonds in the sugar units, leading to the cleavage of the glycosidic bond. Creating LPMO mimics facilitates the study of the mechanism of action, the characterisation of the reactive species responsible for the C─H bond activation and the potential scale up for industrial application. Here, we report the design, structural and functional characterisation of two novel Cu‐binding β‐hairpin peptides, called HisPins, that mimic the Histidine‐brace site of LPMOs. Activity assays were conducted with p‐nitrophenyl‐β‐d‐glucopyranoside (PNPG) and demonstrate that the Cu‐HisPins show LPMO‐like activity on the model substrate. Furthermore, the Cu‐HisPins can also perform light‐driven oxidation of phosphoric acid swollen cellulose (PASC) in the presence of melanin, similarly to some LPMO enzymes. Hence, the Cu‐HisPins represent the first structural and functional LPMO mimics based on short, folded peptide sequences and show a light‐stimulated melanin‐mediated oxidative activity, which is unreported for any LPMO mimic characterised so far. Thus, this work paves the way to further exploration of LPMO mechanism, structure‐activity relationship and substrate scope expansion.

## Graphical abstract

In this work, the copper‐binding histidine brace (His‐brace) motif found in lytic polysaccharide monooxygenase (LPMO) enzymes was engineered in de novo designed peptides that fold as β‐hairpins (HisPins). Cu‐HisPins complexes showed LPMO‐like activity, including light‐stimulated melanin‐mediated oxidative cellulose degradation. Hence, they represent the first structural and functional LPMO mimics based on folded peptide scaffolds.

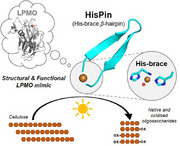

Biofuels (i.e., fuels obtained from biomass) are increasingly sought as an alternative to nonrenewable energy sources. Lignocellulose, the most abundant form of biomass, is a common feedstock for biofuel production.^[^ ^1^ ^]^ However, its mechanical and chemical recalcitrance challenges its industrial use.^[^ ^2^ ^]^

In this context, lytic polysaccharide monooxygenases (LPMOs) play a crucial role in cellulosic biomass conversion. Indeed, LPMOs, classified as auxiliary activity (AA) enzymes in the CAZy database,^[^ ^3^ ^]^ catalyse the oxidative cleavage of β‐1,4‐glycosidic bonds of different polysaccharides (e.g., cellulose and chitin) by oxidising a strong C─H bond (∼100 kcal mol^−1^), forming either C1‐ or C4‐oxidised products (Figure 1a).^[^ ^4^ ^]^

The active site of LPMOs contains a copper ion coordinated in a 3N T‐shaped site by the so‐called “histidine brace” (His‐brace) motif, composed of two His residues: the conserved N‐terminal His chelates the Cu^2+^ ion via its amino group and the N_π_ of the imidazole side chain, whilst a second His binds Cu through the imidazole N_τ_. The coordination sphere of the metal in the resting state is completed by a water molecule (Figure 1b).^[^ ^5^ ^]^

**Figure 1:**
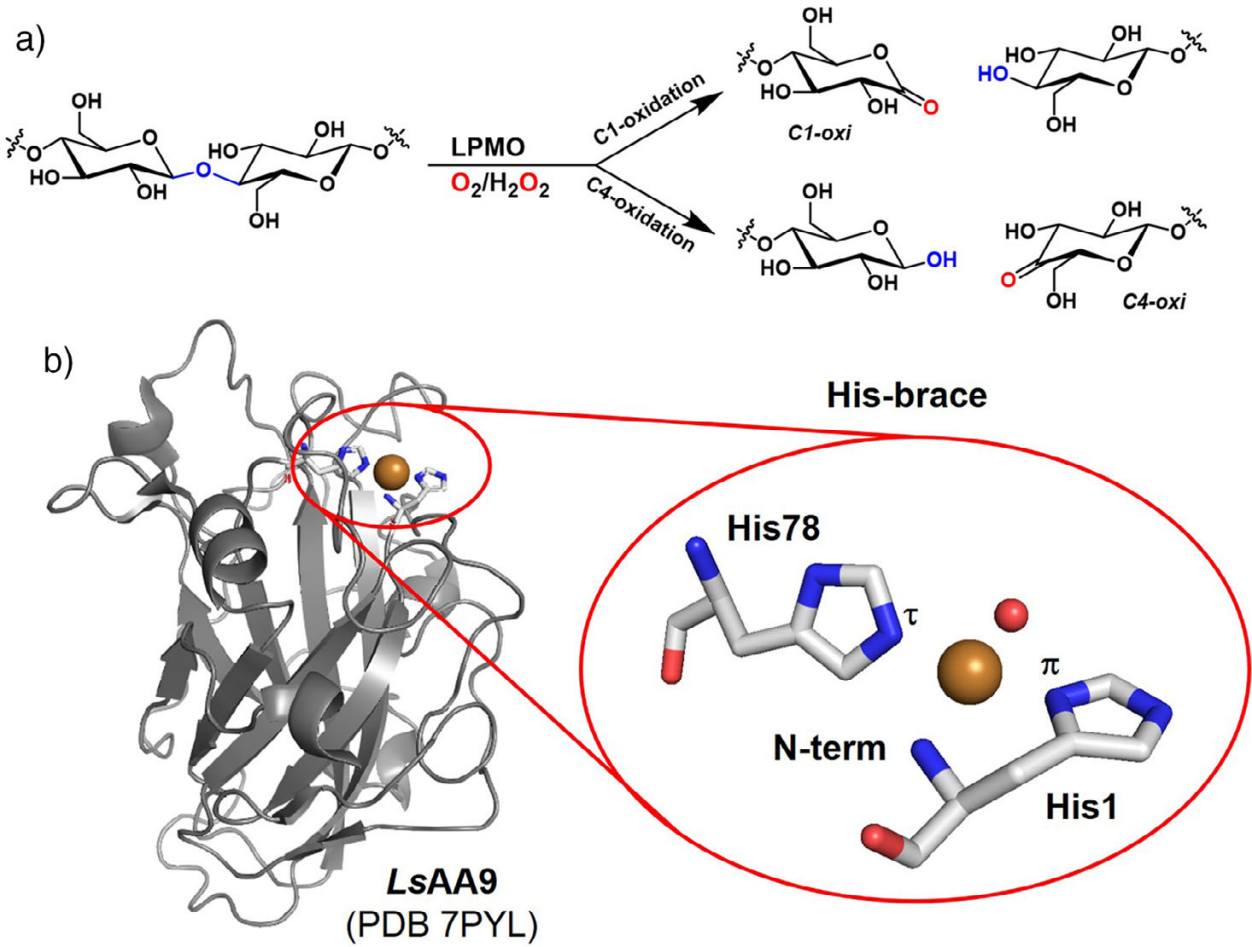
a) Scheme of the reaction catalysed by LPMOs showing both C1‐ and C4‐oxidised products; b) structure of *Ls*AA9 LPMO (PDB 7PYL)^[^ ^6^ ^]^ and its Cu‐binding His‐brace active site. Carbon atoms are in grey, oxygen in red and nitrogen atoms in blue. The Cu ion is represented as a bronze sphere, whilst a coordinated water molecule as a red sphere.

A feature of the His‐brace site that has attracted some attention is the characteristic large “twist angle” between the two best fit planes of the imidazole rings of the histidine side chains (Figure S1). The twist angle measured from reported LPMO crystal structure is significantly larger than in most Cu^2+^ complexes with *trans*‐*N*‐heterocycles (65°–70° compared to less than 30°, respectively), and its potential role in the catalytic cycle is still unclear.^[^ ^7^ ^]^ Furthermore, the *trans* configuration between imidazole rings, and hence between the terminal amine and the co‐substrate (i.e., O_2_ or H_2_O_2_), seems to be crucial for LPMO reactivity, as His‐brace sites bearing *cis*‐imidazole rings (e.g., as in the protein CopC) do not display LPMO‐like activity.^[^ ^8^ ^]^ A reductant (e.g., ascorbate) is required to generate the reduced Cu^+^‐LPMO species that activates O_2_ and/or H_2_O_2_ to produce the oxidised polysaccharides via a mechanism that is still debated.^[^ ^9^, ^10^, ^11^, ^12^, ^13^, ^14^, ^15^, ^16^ ^]^

The interest in elucidating the enzyme mechanism and in establishing novel biocatalysts has prompted the development of LPMO mimics. In the last decade, about a dozen LPMO‐mimicking complexes have been reported, whose activity has been evaluated for a variety of model substrates, spanning from *p*‐nitrophenyl‐β‐d‐glucopyranoside (PNPG) and benzyl alcohol, to cellobiose and polysaccharides such as cellulose and chitin, and under different conditions (e.g., pH and solvents).^[^ ^17^, ^18^, ^19^, ^20^, ^21^, ^22^, ^23^, ^24^, ^25^ ^]^

Early published work focused on easily‐synthesised, small tridentate chelators containing amine, (benzo)imidazole or pyridine N‐donor ligands.^[^ ^18^, ^21^, ^24^ ^]^ Whilst containing the 3N coordination found in the His‐brace, these mimics did not always reproduce the structural features of LPMOs and demonstrated only limited catalytic activity and stability. Among small molecule mimics, the complex reported by Fukatsu et al. stands out as a good structural model of the His‐brace, although it showed only hydrolytic, rather than oxidative, cleavage of PNPG.^[^ ^19^ ^]^ More recently, the short His‐Pro‐His (HPH) peptide sequence, with either l‐ or d‐His in the third position, was explored as a model of the LPMO active site.^[^ ^17^, ^20^ ^]^ Thorough speciation studies of these Cu^2+^‐peptide complexes revealed that they form predominantly dimeric species at physiological pH, whereas a monomeric LPMO‐like species is minor at any pH.^[^ ^20^ ^]^ These studies, alongside the small number of “His‐brace‐like” complexes reported to date, highlight the challenges in designing and synthesising scaffolds able to reproduce the structure and function of the LPMO active site. Recently, a His‐brace site was engineered on the well‐folded azurin protein scaffold, which showed catalytic oxidative cleavage of PNPG as well as cellulose and starch, hence demonstrating that the activity of the His‐brace is not inherently linked to the folding within LPMOs, but can be translated into other structured scaffolds.^[^ ^26^ ^]^

In this context, we report herein the de novo design of peptide sequences folding into β‐hairpins and forming a His‐brace‐like Cu^2+^ binding site, as demonstrated by a combination of spectroscopic and computational studies, and showing LPMO‐like activity. Furthermore, we show that these complexes can oxidatively cleave cellulose in the presence of melanin under light irradiation, as previously observed with the natural enzymes.^[^ ^27^, ^28^, ^29^ ^]^

The de novo design of peptides folding into conformationally stable β‐hairpins and harbouring a His brace motif, hereafter called HisPins (from His‐brace β‐hairpins), was based on a ubiquitin‐derived 16‐residue peptide reported by Searle et al., containing a three‐residue (PDG) bulged turn.^[^ ^30^, ^31^ ^]^ In particular, hydrophilic and hydrophobic residues were alternated along the sequence, except for the turn, in order to create one hydrophilic and one hydrophobic face in the HisPins. An N‐terminal His and a second His residue opposite to the first were introduced to install the His‐brace motif into the β‐hairpin. Following these principles, we designed a 16‐residue peptide, HisPin16 (HIYVKNPDGTEITLHG), where the His‐brace points towards the hydrophilic face (*blue* in Figure 2) of the β‐hairpin, and a 18‐residue peptide, HisPin18 (HSIYVKNPDGTEITLQHG), in which the His‐brace motif is located on the hydrophobic face (*orange* in Figure 2), as validated by AlphaFold2^[^ ^32^ ^]^ structure predictions (Figure 2, *bottom*). These two scaffolds could provide an insight into the effect of the environment around the His‐brace for substrate binding and activity, given the importance of substrate binding for on‐pathway LPMO activity.^[^ ^33^, ^34^ ^]^ The HisPins were synthesised as described in the Supporting Information.

**Figure 2:**
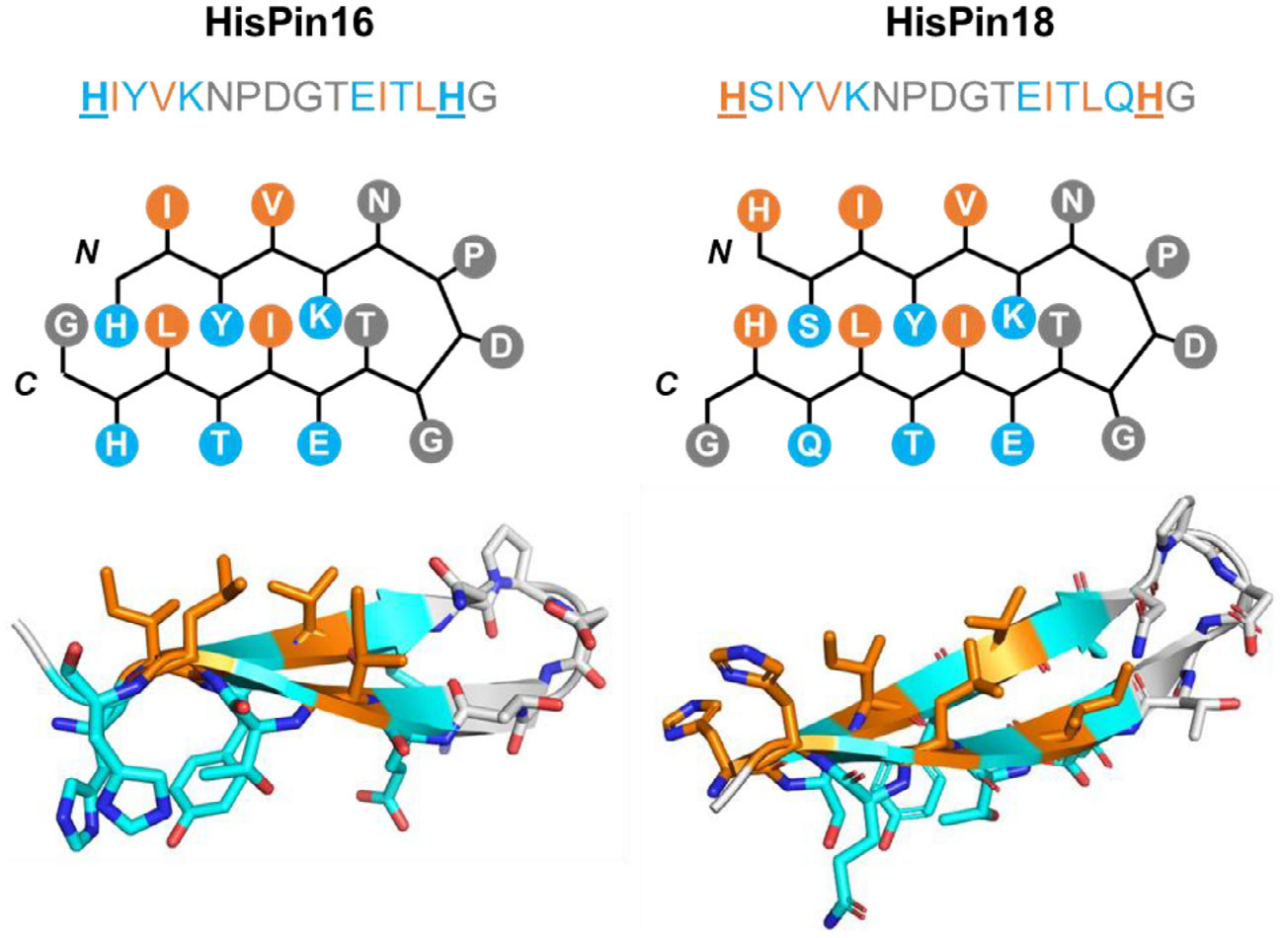
Sequence, schematic structure and AlphaFold2^[^ ^32^ ^]^ models (prepared using PyMol^[^ ^35^ ^]^) of HisPin16 (*left*) and HisPin18 (*right*) peptides. The hydrophilic face of the HisPin peptides is shown in blue, whilst the hydrophobic face is shown in orange. The PDG turn with its flanking N and T residues, as well as the C‐terminal G, are shown in grey.

The secondary structure adopted by the HisPin peptides in solution was examined in the absence and presence of Cu^2+^ by far‐UV circular dichroism (CD). Of note, β‐hairpin peptides do not always show the well‐established CD signature of β‐sheet rich proteins. On the contrary, they exhibit weak CD signals which often are, at first glance, hardly distinguishable from random coil or even α‐helical conformations.^[^ ^36^, ^37^, ^38^ ^]^ This is also the case here, where the CD spectra of the HisPins follow this trend both in the absence and presence of Cu^2+^ (Figure 3a,b), consistent with previously reported peptides bearing a PDG turn.^[^ ^31^ ^]^ The BeStSel algorithm was used to carry out a CD‐based secondary structure determination.^[^ ^39^, ^40^ ^]^ All spectra were fitted by a majority (50–60%) of antiparallel β‐strand and turns, along with disordered (“others”) secondary structure elements (Figure 3c and S3 and Table S1). These results are commensurate with the dynamic folding of the HisPins as β‐hairpins in solution. The CD data also demonstrate that the HisPins assume a β‐hairpin folding that is independent of the addition of Cu^2+^. ATR‐FTIR (Attenuated Total Reflectance‐Fourier Transform Infrared) spectra were also recorded (Figure 3d,e) to further assess the formation of β‐hairpins, as the amide I band (1600–1700 cm^−1^) is sensitive to the secondary structure adopted by proteins and peptides.^[^ ^41^ ^]^ Both HisPin16 and HisPin18, with and without Cu^2+^, show very similar amide I bands with a maximum at about 1630 cm^−1^ and a tail that extends up to about 1680–1690 cm^−1^. These features are characteristic of antiparallel β‐sheets,^[^ ^42^ ^]^ including previously reported β‐hairpin peptides with a PDG turn.^[^ ^43^, ^44^ ^]^ Overall, the combination of CD and IR data suggests that the HisPins are folded as β‐hairpins and that the secondary structure conformation is determined solely by the peptide sequence, providing a pre‐arranged coordination environment for the metal ion.

**Figure 3:**
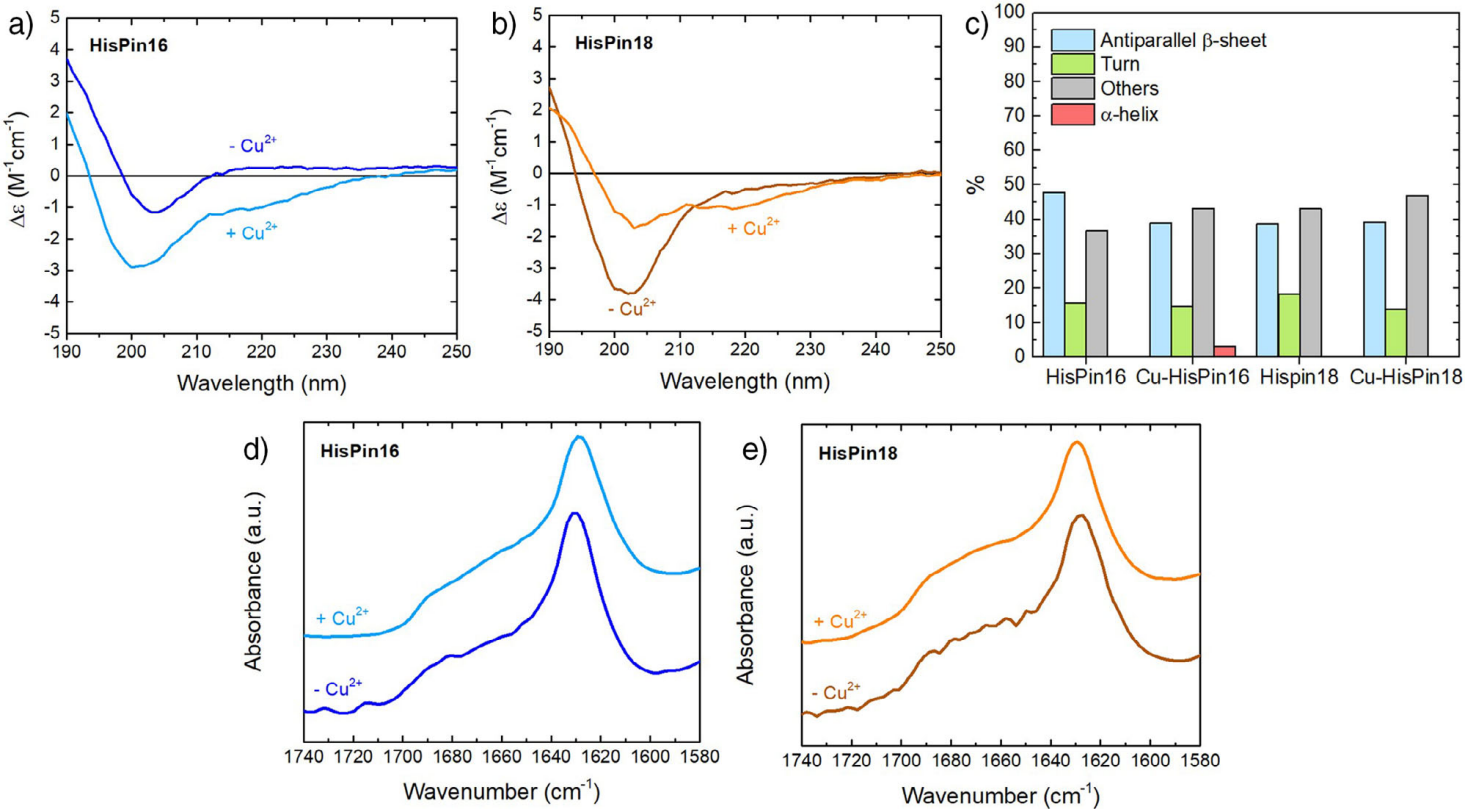
Secondary structure characterisation by CD (panels a–c) and ATR‐FTIR (panels d and e) of HisPin16 (dark blue traces in panels a and d), Cu‐HisPin16 (light blue traces in panels a and d), HisPin18 (brown traces in panels b and e) and Cu‐HisPin18 (orange traces in panels b and e). c) Fits of CD spectra using the BeStSel algorithm^[^ ^39^, ^40^ ^]^ (blue, *antiparallel β strand*; green, *turn*; red, *α‐helix*; grey, *others*). In panels d and e, spectra are stacked. Conditions: for CD, 50 µM HisPins, 50 µM CuSO_4_ in 5 mM phosphate buffer (PB) pH 7.4; for ATR‐FTIR, 1.2 mM HisPins, 1 mM CuSO_4_ in 100 mM PB pH 7.4.

The binding of Cu^2+^ to HisPins was investigated by continuous wave (CW) X‐band electron paramagnetic resonance (EPR) spectroscopy at low temperature (77 K). The EPR spectra of the Cu‐HisPin16 and Cu‐HisPin18 complexes (Figure 4a,b) are virtually identical and show the presence of a single Cu species. The spin‐Hamiltonian parameters obtained by the simulation of the spectra (*g*_3 _≈ 2.27, |A_3_| = 535 MHz, Table S2) are typical of a Type‐2 Cu^2+^ site with a square planar 3N1O coordination sphere. They lie well within the regions of the Peisach–Blumberg plot where most axial‐type AA9‐LPMOs are found (Figure 4c and Table S3),^[^ ^45^ ^]^ and are similar to those reported for the AA9 from *Lentinus similis* (*Ls*AA9) bound to cellohexaose (Cello_6_).^[^ ^46^ ^]^ Given the extensive overlap between the 3N1O and 2N2O coordination parameters in the Peisach–Blumberg plot,^[^ ^47^ ^]^ it is not possible to definitely discriminate between the two sets of ligands solely on the basis of the EPR, and hence, the HisPins might be coordinating the Cu^2+^ via the N‐terminal His alone. As a control, the EPR spectrum of Cu^2+^ bound to the bidentate histidine methyl ester (His^OMe^) ligand in 1:1 stoichiometric ratio exhibited different EPR parameters (*g*_3 _= 2.295, |A_3_| = 520 MHz, Table S3), further supporting the involvement of the distal C‐terminal His residue in the coordination sphere of the metal ion.

**Figure 4:**
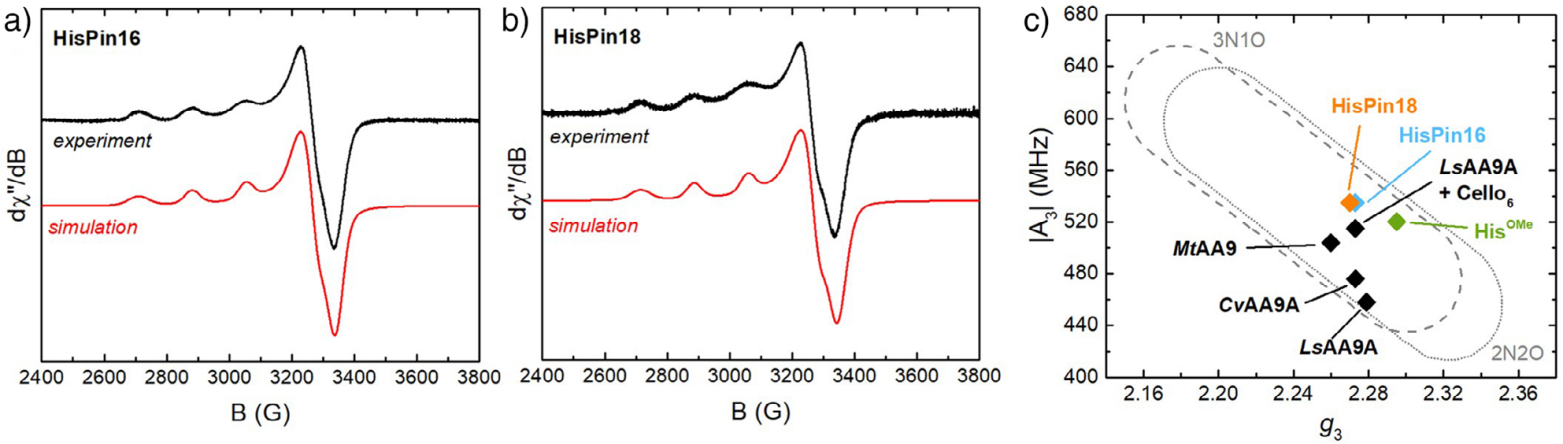
Low‐temperature (77 K) EPR spectra of Cu‐HisPin16 a) and Cu‐HisPin18 b) and Peisach–Blumberg plot^[^ ^47^ ^]^ showing the EPR parameters (*g*_3_ and |A_3_|) of the Cu‐HisPins compared to Cu‐His^OMe^ and some AA9‐LPMOs c), values plotted are reported in Table S3). Conditions: 0.25 mM HisPins, 0.2 mM CuSO_4_ in 100 mM PB pH 7.4, 10% *v/v* glycerol.

To further investigate the structure of the Cu^2+^ binding site in HisPin peptides, we used ab initio molecular dynamics (AIMD), with the GFN2‐*x*TB density functional tight‐binding method.^[^ ^48^ ^]^ Initial calculations narrowed down the appropriate structures to those including an explicit water molecule binding to Cu^2+^, along with His1 bound via the N‐terminal amino group and N_π_ of the side chain, and His15/17 bound via either N_π_ (Cu‐HisPin‐N_π_) or N_τ_ (Cu‐HisPin‐N_τ_) (Table S4). For Cu‐HisPin16, in both N_π_ and N_τ_ variants, the amino group lies *trans* to the water molecule, as found in LPMOs. However, only the N_τ_ variant shows a distorted square‐planar geometry, whilst the N_π_ variant is nearly tetrahedral, as evidenced by the calculated geometry index τ_4_^[^ ^49^ ^]^ (see Supporting Information and Table S4). Hence, based on the observed axial EPR spectrum, only the Cu‐HisPin16‐N_τ_ variant seems to contribute to the speciation of the complex (Figure 5). In the case of Cu‐HisPin18, the N_τ_ variant features a geometry close to square planar (Figure 5 and Table S4), with the imidazole rings found *trans* to each other. The N_π_ variant, instead, shows a penta‐coordinated distorted square pyramidal geometry (Figure 5 and Table S4) in which the two imidazole rings are *cis* and the 3N ligands lie on the equatorial plane together with an O atom from the carbonyl of His17, whilst a water molecule binds axially. Despite their different coordination geometries, both these variants are in principle compatible with the EPR spectrum recorded, and hence, both models are considered for Cu‐HisPin18.

**Figure 5:**
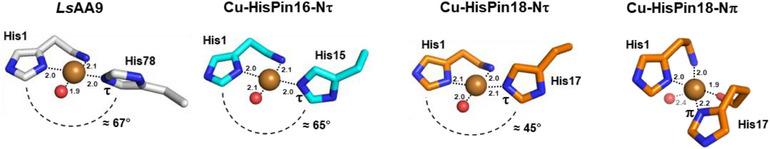
Structure and key metric parameters of Cu^2+^‐binding sites in *Ls*AA9 (PDB 7PYL),^[^ ^6^ ^]^ Cu‐HisPin16 and Cu‐HisPin18. For the HisPins, the structures shown are representative snapshots from AIMD simulations with average geometry index and twist angle between the imidazole rings.

An analysis of the structural parameters for the N_τ_ variants, which are most similar to the native His‐brace site in axial‐type LPMOs, shows that the twist angle between the imidazole rings of His1 and His15/17 (calculated for snapshots throughout the second half of the simulation and for which *τ*_4_ < 0.5) is ≈ 65° for Cu‐HisPin16 and ≈ 45° for Cu‐HisPin18 (Figure 5). Hence, Cu‐HisPin16 seems to be a better structural model for LPMOs than Cu‐HisPin18.

The small twist angle found in the majority of LPMO mimics reported to date, with the exception of the bis‐imidazole complex reported by Fukatsu et al.^[^ ^19^ ^]^ in which a constrained linker causes the twist, has led to the hypothesis that steric requirements and interactions with other residues in the proximity of the active site are necessary to aid and/or maintain the nonplanar arrangement of the rings in the enzyme. The Cu‐HisPins naturally adopt this conformation in the absence of such constraints, which is a striking difference compared to the other LPMO mimics and suggests that this is an inherent feature of the His‐brace.

Having assessed the structural and electronic properties of the Cu‐HisPin complexes, their LPMO‐like activity was first evaluated using PNPG, a model substrate that has been widely used in the literature to assess the activity of LPMO mimics.^[^ ^18^ ^]^ This assay consists in the oxidative cleavage of PNPG in the presence of Cu^2+^ or a Cu^2+^‐complex and H_2_O_2_, with the formation of *p*‐nitrophenolate (PNP), which can be easily monitored by UV–vis spectroscopy at 400 nm, and gluconolactone (Figure 6a). Remarkably, PNP generation appeared to be ca. 44‐ and 32‐fold faster with Cu‐HisPin16 and Cu‐HisPin18, respectively, than CuSO_4_, and ca. 10‐fold faster than the Cu‐His^OMe^ complex (Figure 6b,c). Moreover, PNPG cleavage by Cu‐HisPin16 was ca. 1.4‐fold faster than by Cu‐HisPin18. The formation of oxidised products was confirmed through the detection of gluconate, the hydrolytic product of gluconolactone (Figure 6a), by HPAEC‐PAD (High‐Performance Anion‐Exchange Chromatography with Pulsed Amperometric Detection, Figure 6d) and ESI‐MS (Electrospray Ionisation‐Mass Spectrometry, Figure S4). The HPAEC‐PAD analysis indicated that a higher amount of gluconate was produced by Cu‐HisPin16 than Cu‐HisPin18, corroborating the results obtained by UV–vis detection of PNP. The spectrophotometric assays showed that PNP formation with the Cu‐HisPin complexes reached a plateau within about 1 h under our experimental conditions. This is likely due to the inactivation of the Cu‐HisPin complexes through oxidative damage of the peptides by excess H_2_O_2_, a well‐known phenomenon for natural LPMOs.^[^ ^50^ ^]^ Nevertheless, these data showed that the Cu‐HisPin complexes can reproduce LPMO activity and that the β‐hairpin scaffold has a clear impact on the catalytic action of the Cu centre. We also tested the ability of Cu‐HisPin complexes to degrade extended polysaccharides using phosphoric acid swollen cellulose (PASC) as the substrate and ascorbate as the reductant, which lead to no product formation. Nevertheless, the formation of native as well as both single (C1‐ or C4‐oxi) and mainly double (C1/C4‐oxi) oxidised oligosaccharides was observed by HPAEC‐PAD analysis upon addition of melanin, a photosensitiser known to trigger LPMO activity, in the presence of light (Figure 6e,f and Figure S5).^[^ ^28^ ^]^ Melanin photosensitisation leads to the photoreduction of dioxygen to hydrogen peroxide and the concomitant reduction of Cu^2+^ to Cu^+^.^[^ ^28^ ^]^ Unlike LPMO enzymes, which typically have a unique pattern of native and oxidised oligosaccharides released from insoluble polysaccharide,^[^ ^51^ ^]^ the oligosaccharides produced by Cu‐HisPins did not show a specific ratio between native and oxidised species, or any pattern regarding the size distribution relative to their oxidation. This suggests that Cu‐HisPins act randomly on the polymer surface oxidising the glycosidic bond without the constraint imposed by the enzyme structure (including substrate interaction, enzyme steric conformation and arrangement).

**Figure 6:**
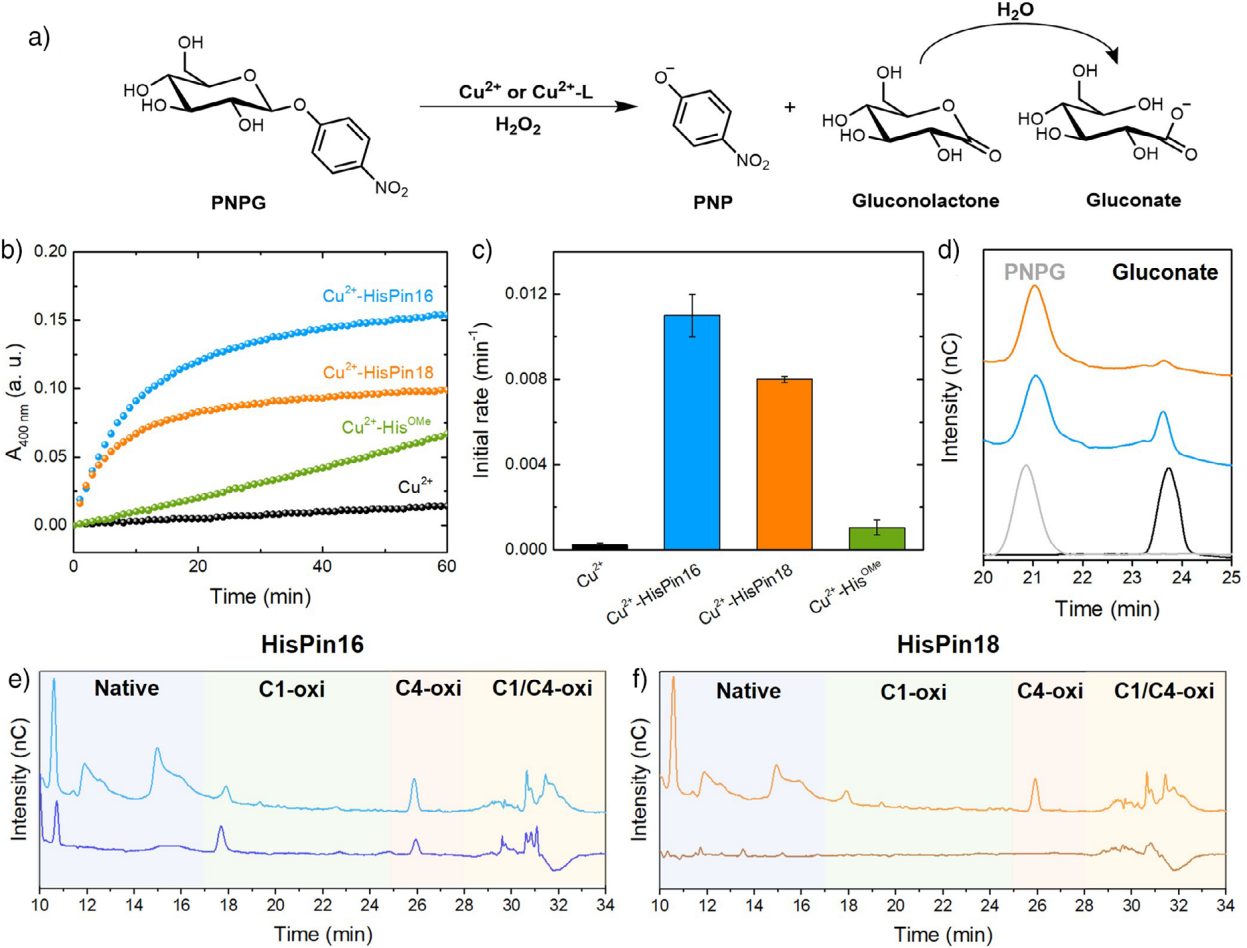
LPMO‐like activity of Cu‐HisPin peptides: oxidative cleavage of PNPG (a–d) and light‐stimulated melanin‐mediated oxidative cleavage of PASC (e–f). a) Reaction scheme of oxidative cleavage of PNPG; b) Kinetics of PNP formation catalysed by Cu‐HisPin16 (blue), Cu‐HisPin18 (orange), Cu‐His^OMe^ (green) and CuSO_4_ (black) followed by UV–vis absorption at 400 nm; c) Initial rates for reactions described in b; d) Normalised HPAEC‐PAD chromatograms of PNPG (grey) and gluconate (black) reference solutions and of the reaction mixture containing PNPG, H_2_O_2_ and Cu‐HisPin16 (blue) or Cu‐HisPin18 (orange); e) HPAEC‐PAD chromatograms of PASC incubated with Cu‐HisPin16 in the presence of ascorbate and melanin in the dark (dark blue) or under visible light irradiation (light blue); f) HPAEC‐PAD chromatograms of PASC incubated with Cu‐HisPin18 in the presence of ascorbate and melanin in the dark (brown) or under visible light irradiation (orange). In panels e and f, HPAEC‐PAD chromatograms of matched control mixtures containing Cu^2+^, ascorbate, PASC and melanin in the dark or under light irradiation were subtracted (Figure S4). Conditions: b–d) 120 µM HisPins, 100 µM CuSO_4_, 1 mM PNPG, 1 mM H_2_O_2_ in 100 mM PB at pH 7.4; e–f) 0.5% (w/v) PASC was incubated with 20 µM Cu‐HisPins, 20 µM CuSO_4_, 1 mM ascorbate, 0.1 mM melanin in 20 mM PB pH 7.4.

Similarly to the results obtained with PNPG, Cu‐HisPin16 showed a higher activity than Cu‐HisPin18 on PASC (Figure 6e–f). We speculate that this difference, which warrants further investigations, may arise from one or more of the following structural factors. Based on AIMD simulations, the Cu‐binding site in HisPin16 is very similar to the His‐brace site in LPMOs, whilst HisPin18 could also form a square pyramidal site that is not expected to have LPMO‐like activity due to the different arrangement of the ligands around the metal centre, namely the *cis* conformation of the imidazole rings and the terminal amine no longer in *trans* position to the reactive oxygen site. Secondly, although the role of the twist angle in LPMOs has not been elucidated, the lower twist angle observed in the His‐brace of HisPin18 relative to HisPin16 may also influence the catalytic cycle and be responsible for the lower activity and faster deactivation. Finally, in Cu‐HisPin16, the His‐brace points towards the hydrophilic face of the hairpin, which might favour binding to the sugar moieties by H‐bond interactions.

In conclusion, we report the de novo design of short peptide sequences folding into amphipathic β‐hairpins and incorporating a LPMO‐like His‐brace Cu^2+^ binding site. Both Cu‐HisPin complexes showed LPMO‐like activity towards the widely used model substrate PNPG. Whilst comparisons with the literature need care due to the variety of experimental conditions utilised and parameters reported in the PNPG assay, the Cu‐HisPins are broadly similar to early LPMO mimics and the Cu‐HPH peptides,^[^ ^18^, ^20^ ^]^ but considerable optimisation must be done to achieve the performance of other mimics or the enzyme. Notably, the Cu‐HisPins were also able to oxidatively cleave cellulose, a natural LPMO substrate, in the presence of melanin as photosensitiser. Such light‐stimulated melanin‐mediated activity has been observed with LPMOs, but, to the best of our knowledge, the Cu‐HisPins represent the first examples of photoactivated LPMO mimics. The advantage of such light‐driven reactions is linked to the possibility of controlling the kinetics of the H_2_O_2_ co‐substrate generation at sub‐lethal doses for enzymes and HisPins by tuning light irradiation and melanin concentration. Of note, in both PNPG and PASC cleavage, the Cu^2+^ complex of HisPin16 outperformed the analogous complex with HisPin18. Based on AIMD simulations, this difference can be attributed to the higher structural similarity of Cu‐HisPin16 to LPMOs. Hence, the Cu‐HisPin complexes reported herein are the first structural and functional models of the LPMO His‐brace motif based on short well‐folded peptides, opening the way to further mechanistic investigations of LPMOs. Furthermore, the β‐hairpin scaffolds give easy access to a variety of synthetic variations to further explore structure‐activity relationships and expand substrate scope.

## Supporting Information

Methods, supplementary data and additional references [39, 40, 45] are included in the Supporting Information (SI).

## Author Contributions

L.C. conceived the study. E.F., D.C. and D.R. contributed to the experimental design. E.F., R.T., E.T. and D.C. performed the experiments and analysed the data. D.R. performed molecular dynamics simulations. D.R. and E.F. analysed molecular dynamics simulations. E.F., D.C., D.R. and L.C. wrote the manuscript. All authors have read and contributed to the preparation of the manuscript.

## Conflict of Interests

The authors declare no conflict of interest.

## Supporting information



Supporting Information

## Data Availability

The data that support the findings of this study are available from the corresponding author upon reasonable request.
