# Supplementary material for: De Novo Designed β‐Hairpin Peptides Mimicking the Copper‐Binding Histidine Brace Motif of Lytic Polysaccharide Monooxygenases
Source: Angew Chem Int Ed Engl. 2025 Aug 15;64(40):e202513990. doi: 10.1002/anie.202513990 (PMC12462749; doi:10.1002/anie.202513990)
Supplement: Supplementary file 1 — Supporting Information [file ANIE-64-e202513990-s001.docx]

**Supplementary Information**

***De Novo* Designed β-Hairpin Peptides Mimicking the Copper-Binding Histidine Brace Motif of Lytic Polysaccharide Monooxygenases**

Enrico Falcone,^1†^ Rosemary Tomey,^1^ Emma Turley,^1^ David Cannella,^2^ David Robinson^3^ and Luisa Ciano^1^*

^1^ *School of Chemistry, University of Nottingham, University Park, Nottingham NG7 2RD, UK*

^2^ *PhotoBiocatalysis Unit, Biomass Transformation Lab – BTL, and Crop Production Biostimulation Lab – CPBL, Universitè Libre de Brussels, ULB, Brussels, Belgium*

^3^ *Department of Chemistry and Forensics,* *School of Science and Technology, Nottingham Trent University, Nottingham, NG11 8NS, UK*

^†^current address: LCC-CNRS, Université de Toulouse, CNRS, Toulouse, France

*corresponding author: luisa.ciano@nottingham.ac.uk

**Table of contents**

**Methods**…………………………………………………………………………………………….2

**Supplementary Figures**……………...…………………………………………………………..5

**Supplementary Tables**……...……………………………………………………………………8

**Methods**

***Materials***

Commercially available chemicals were used without further purification. All stock solutions were prepared in Milli-Q water. The concentration of peptide solution was determined by tyrosine UV-vis absorption (Δε_276-296_ = 1410 M^−1^cm^−1^).^[52]^ Phosphate buffer (PB, 500 mM, pH 7.4) was prepared by mixing KH_2_PO_4_ and K_2_HPO_4_ and adjusting the pH with KOH. The concentration of CuSO_4_·5H_2_O solution was verified by UV-Vis spectroscopy (ε_780_ = 12 M^−1^ cm^−1^). A fresh PNPG solution in ultrapure water was prepared daily. PASC and L-DOPA melanin from *Aspergillus nidulans* A773 harvested after 20 days incubation in liquid medium and 12 hours light and 12 hours darkness photoperiod were prepared as previously reported by Cannella *et al*.^[27]^ and Monclaro *et al*.,^[28]^ respectively.

***Solid-phase peptide synthesis***

Standard Fmoc/^t^Bu solid-phase peptide synthesis was carried out using an Initiator+ Alstra™ (Biotage®) automated microwave-assisted synthesiser. Peptides were synthesised on a Rink Amide MBHA resin HL (Novabiochem®) in a 0.2 mmol scale. Couplings were performed using 4 equivalents of Fmoc-protected amino acids and DIC/Oxyma (0.5 M) as coupling agents in DMF. Microwave heating (5 min, 75 °C) was used for the coupling of all amino acids but His (60 min, room temperature). Capping of the unreacted amine group was carried out using 5% acetic anhydride and 10% DIEA in DMF. N-terminal Fmoc deprotection was carried out using 20% piperidine in DMF supplemented with 5% formic acid to minimise aspartimide formation.^[53]^ Resin cleavage and side-chain deprotection were performed by treatment with 95% TFA, 2.5% H_2_O and 2.5% TIS for 4h. The crude peptides were precipitated with cold ether and purified by HPLC on a C18 column (Waters™ Sunfire® Prep C18 OBD, 19 mm x 150 mm, pore size 100 Å, particle size 5 µm). Purified peptides were analysed by analytical HPLC and ESI-MS ([HisPin16+H]^+^: experimental *m/z* = 1793.9246, calculated *m/z* = 1793.9180; [HisPin16+2H]^2+^: experimental *m/z* = 897.4754, calculated *m/z* = 897.4626; [HisPin16+3H]^3+^: experimental *m/z* = 598.3288, calculated *m/z* = 598.6442; [HisPin18+2H]^2+^: experimental *m/z* = 1005.0152, calculated *m/z* = 1005.0080; [HisPin18+3H]^3+^: experimental *m/z* = 670.3472, calculated *m/z* = 670.3411). Yields: HisPin16, 16%; HisPin18, 13%.

***CD spectroscopy***

CD spectra were recorded in a 0.1 cm path quartz cuvette using a Chirascan (Applied Photophysics) spectrometer. Three spectra were averaged for each sample (containing 50 μM peptide in 5 mM PB pH 7.4 with or without 50 μM CuSO_4_) and the spectrum of the blank (buffer) was subtracted after the sample measurement. CD spectra were fitted using the BeStSel method.^[39-40]^

***ATR-FTIR spectroscopy***

ATR-FTIR spectra were obtained using an Alpha (Bruker) spectrometer by accumulating 64 scans with a spectral resolution of 4 cm^−1^. To avoid interference by TFA, exchange of peptide TFA counterions with chloride was carried out by three cycles of dissolution of the peptide in 0.1 M aqueous HCl followed by lyophilization. The samples (10 μL, 1.2 mM peptide in 100 mM PB pH 7.4 with or without 1 mM CuSO_4_) were deposited on the ATR crystal and left to dry for a few minutes.

***EPR spectroscopy***

Continuous-wave X-band EPR spectra of Cu-HisPin16 and Cu-HisPin18 at 77 K were acquired on a Bruker EMX spectrometer operating at ∼9.40 GHz. For each spectrum, 8 scans were accumulated with modulation amplitude of 4 G, modulation frequency of 100 kHz and microwave power of 10.00 mW. Samples (0.2 mM CuSO_4_ and 0.25 mM peptide in 100 mM phosphate buffer pH 7.4) were supplemented with 10% glycerol as a glassing agent before flash freezing in liquid N_2_ and introduction in a finger Dewar pre-filled with liquid N_2_. Spectra simulations were carried out using EasySpin integrated into MATLAB R2014a.^[54]^ It was assumed that *g* matrix and A tensors were axially coincident. Exact parameters for the perpendicular region could not be obtained from X-band alone, although it was noted that good simulations of the experimental data were achieved with the parameters reported in **Table S2**. Furthermore, it was also noted that the simulations were improved by the addition of two coupled nitrogen nuclei, although the exact value of the coupling could not be determined given the lack of well resolved superhyperfine coupling. Hence, the values reported in **Table S2** have to be considered as a mere indication of the magnitude.

***Ab initio molecular dynamics***

Initial structures for the density functional theory (DFT) calculations were taken from the top ranked predicted structure from AlphaFold2 for both HisPin16 and HisPin18 peptides. A Cu^2+^ ion was added to the structure along with a single explicit water molecule. The structures were minimised using the Universal Force-Field (UFF). These starting structures were then used to start an *ab initio* molecular dynamics (AIMD) simulation using the GFN2-xTB semi-empirical tight-binding^[48]^ Hamiltonian within the density functional tight binding (DFTB) formalism. Equilibration was performed for 5000 steps with a timestep of 0.5 fs, using initial velocities sampled from a distribution at 300 K. The Nosé-Hoover chains (NHC) thermostat was used, with a temperature of 298 K and a coupling constant of 10 fs. Production dynamics employed a timestep of 1.0 fs and ran for 10000 steps (10 ps). The analytical linearised Poisson-Boltzmann (ALPB) solvation model, using water as the solvent, was employed throughout. Structures were written to file every 5 fs. The N- and C-terminal residues were neutral; aspartic acid and glutamic acid were negative (formal -1 charge) and lysine was positive (formal +1 charge).

For each structure in the trajectory file, we calculated the geometry index τ_4_ or τ_5_:^[49, 55]^

$$\tau_{4}\text{ = }\frac{\text{360° - }\left( \text{α + β} \right)}{\text{141°}}$$

$$\tau_{5}\text{ = }\frac{\left( \text{β}\text{ - }\text{α} \right)}{\text{60°}}$$

where $\alpha$ and $\beta$ ($\beta$ > $\alpha$) are the two largest bond angles of the copper coordination site, and a value of $\tau_{4}=0$ corresponds to a perfect square planar structure and $\tau_{4}=1$ corresponds to a perfect tetrahedral structure, while $\tau_{5}=0$ corresponds to a perfect square pyramidal geometry and $\tau_{5}=1$ to a perfect trigonal bipyramidal geometry.

***PNPG assay***

The assay was carried out in 96-well microplates and the absorbance of the *p*-nitrophenolate (λ = 400 nm) was measured using a Biotek Epoch2 microplate reader. Reaction mixtures contained 100 μM CuSO_4_, 120 μM peptide, 1 mM PNPG and 1 mM H_2_O_2_ in 100 mM PB at pH 7.4.

***Light-stimulated melanin-mediated PASC cleavage***

LPMO-like activity on PASC was assessed by adapting a method reported previously.^[28]^ Briefly, 0.5% (w/v) PASC was incubated with 20 μM Cu-HisPin complex (or CuSO_4_ as a control), 1 mM ascorbate as the reductant and 0.1 mM melanin in 20 mM PB pH 7.4. Reaction mixtures were kept shaking under visible light (210 μmol photons/m^2^/s) or darkness. After overnight incubation, samples were centrifuged at 15000 *g* for 3 min.

***HPAEC-PAD***

Native and oxidised oligosaccharides formed upon PASC incubation with Cu-HisPin complexes were analysed by high-performance anion-exchange chromatography (HPAEC), performed on an ICS-6000 system (Dionex, Sunnyvale, CA, USA), equipped with a pulsed amperometry detection (PAD) and with a CarboPac PA1 column (2 × 50 mm guard column followed by a 2 × 250 mm analytical column), operated at a flow of 0.25 mL min^−1^, at 30 °C. The analysis of native, C1-oxidised, C4-oxidised, and double C1/C4-oxidised oligosaccharides was conducted using a gradient elution profile with a mix of NaOH and sodium acetate, as described by Cannella *et al*.^[56]^ The separation of PNPG and gluconate was obtained on the same HPAEC equipment and PA1 column but operated in different conditions. Notably, eluents A (milliQ water) and B (NaOH 1M) were mixed as following: at start the mix was 96:4 (A:B), and kept increasing the B for 18 minutes until 35% (65:35), kept stable for 2 minutes (until 20 minutes), then returned to initial conditions in 30 seconds (96:4, at 20,5 minutes). These conditions were kept for 9,5 minutes until end of run (total time 30 minutes).^[51]^

**Supplementary Figures**


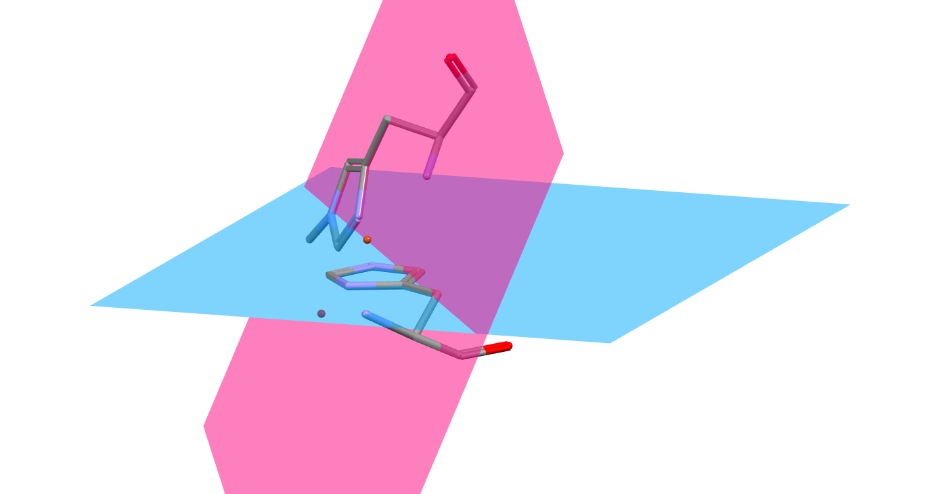

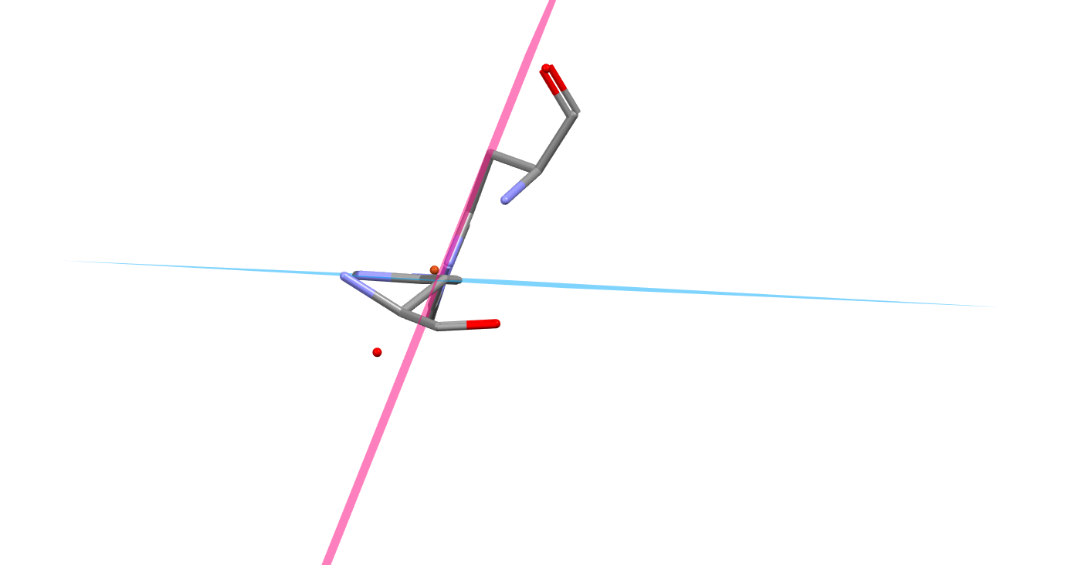


Twist angle

**Figure S1.** Structure of the Histidine brace from *Ls*AA9 LPMO (PDB: 5ACG)^[46]^ showing the two best fit planes of the imidazole rings, highlighting the twist angle between the planes.


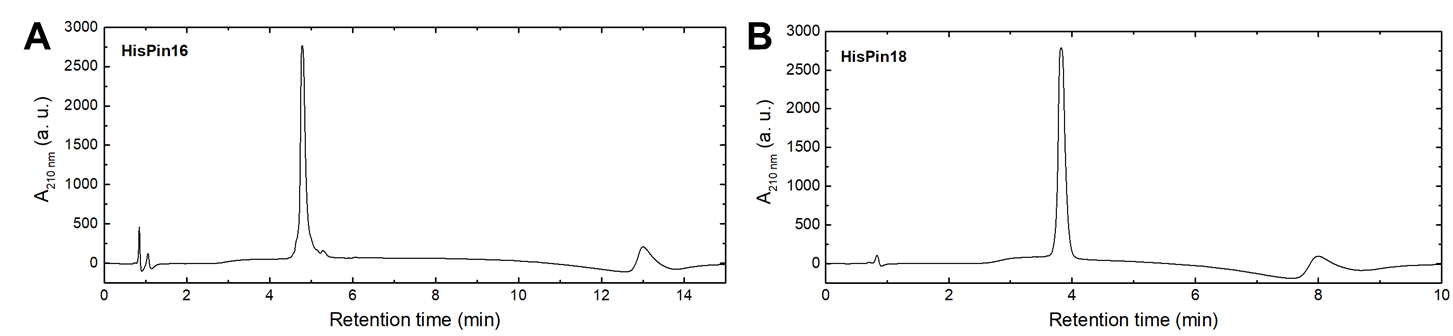


**Figure S2.** HPLC chromatograms of purified HisPin16 (**A**) and HisPin18 (**B**).


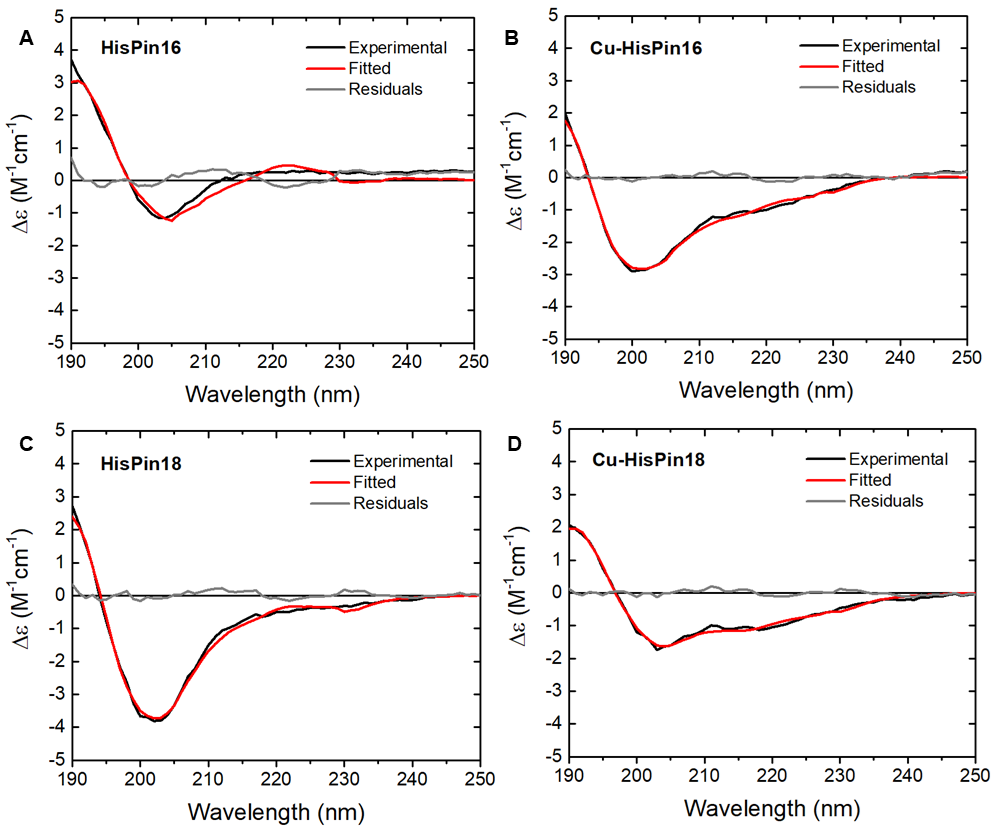


**Figure S3.** Fitting of CD spectra with the BeStSel algorithm:^[39-40]^ **A**) HisPin16, **B**) Cu-HisPin16, **C**) HisPin18, **D**) Cu-HisPin18.

**
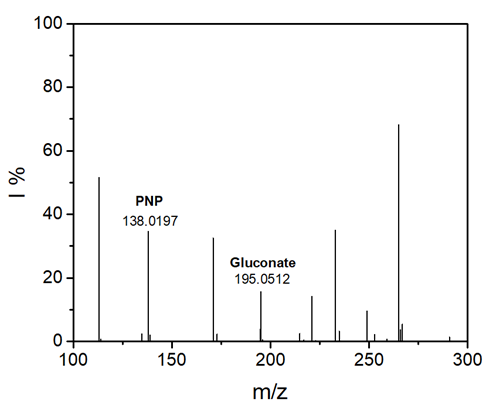
**

**Figure S4.** HR-ESI-MS (negative mode) spectrum of a PNPG assay reaction mixture collected at the end of the reaction, showing the formation of PNP (C_6_H_4_NO_3_, exact mass: 138.0191 Da) and gluconate (C_6_H_11_O_7_, exact mass: 195.0505 Da).

**
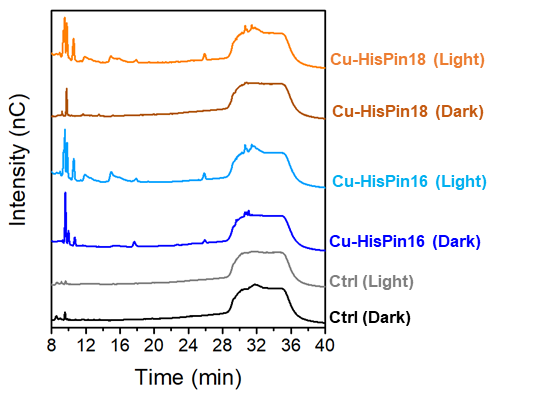
**

**Figure S5.** Raw HPAEC-PAD chromatograms of mixtures containing PASC, Ascorbate and melanin in the presence of Cu-HisPin16 or Cu-HisPin18 or Cu^2+^ (Ctrl), in the dark or under visible light irradiation, in 20 mM phosphate buffer pH 7.4.

**Table S1.** Fit of CD spectra of HisPin16 and HisPin18 with and without Cu using the BeStSel algorithm.^[39-40]^

|  | | **Antiparallel** | **Turn** | **Helix** | **Others** | **RMSD** |
| --- | --- | --- | --- | --- | --- | --- |
| **HisPin16** | **-Cu** | 47.8 | 15.6 | 0 | 36.6 | 0.2196 |
|  | **+Cu** | 38.8 | 14.8 | 3.1 | 43.2 | 0.0906 |
| **HisPin18** | **-Cu** | 38.6 | 18.3 | 0 | 43.1 | 0.1047 |
|  | **+Cu** | 39.2 | 13.9 | 0 | 46.9 | 0.0751 |

**Table S2.** List of EPR spin-Hamiltonian parameters used in the simulation of experimental spectra collected at 77 K for 0.2 mM solution of Cu-HisPin16 or Cu-HisPin18 in 100 mM phosphate buffer pH 7.4 with 10% *v/v* glycerol.

|  |  | **Cu-HisPin16** | **Cu-HisPin18** |
| --- | --- | --- | --- |
| **g values** | *g_1_*  *g_2_*  *g_3_* | 2.055  2.070  2.273 | 2.056  2.070  2.270 |
| **A_Cu_ (MHz)** | \|A_1_\|  \|A_2_\|  \|A_3_\| | 15  42  535 | 30  70  535 |
| **A_N_ (isotropic) (MHz)** |  | 30, 40 | 30, 40 |
| **A_cu_ strains (MHz)** |  | 10, 20, 100 | 80, 80, 100 |
| **Line widths (mT)** |  | 2.5, 2.5 | 2.5, 2.5 |
| **Frequency (GHz)** |  | 9.438579 | 9.436644 |
|  |  |  |  |

**Table S3.** Principal EPR parameters of Cu^2+^ bound to HisPin peptides, His^OMe^ and some AA9-LPMOs.^[45]^

| **Ligand** | ***g*_3_** | **\|A_3_\| (MHz)** |
| --- | --- | --- |
| HisPin16 | 2.273 | 535 |
| HisPin18 | 2.270 | 535 |
| His^OMe^ | 2.295 | 520 |
| *Mt*AA9 | 2.260 | 504 |
| *Cv*AA9A | 2.273 | 476 |
| *Ls*AA9A | 2.279 | 458 |
| *Ls*AA9A + Cello_6_ | 2.273 | 515 |

**Table S4.** Calculated structural parameters for each of the AIMD simulations

| **Complex** | **Variant** | **τ_4_** | **τ_5_** | **Geometry** | **Twist angle** |
| --- | --- | --- | --- | --- | --- |
| **Cu-HisPin16** | N_π_ | 0.71 ± 0.16 | n/a | Tetrahedral | n.d. |
|  | N_τ_ | 0.46 ± 0.17 | n/a | Distorted square planar | ≈ 65° |
| **Cu-HisPin18** | N_π_ | n/a | 0.29 ± 0.17 | Square pyramidal | n.d. |
|  | N_τ_ | 0.34 ± 0.22 | n/a | Distorted square planar | ≈ 45° |
